# Chinese Doctoral Students Involved in Interdisciplinary Learning Score Higher on Scientific Creativity: The Roles of Teamwork Skills and Collaborative Behaviors

**DOI:** 10.3390/bs14111046

**Published:** 2024-11-05

**Authors:** Shuzhen Chen, Lichao Ma, Yinqi Ma

**Affiliations:** 1 Department of Educational Administration and Policy, Faculty of Education, The Chinese University of Hong Kong, Hong Kong 999077, China; shuzhenchen@link.cuhk.edu.hk; 2 Institute of Education, Tsinghua University, Beijing 100084, China; malc21@mails.tsinghua.edu.cn; 3 College of Education, Zhejiang Normal University, Jinhua 321004, China

**Keywords:** interdisciplinary learning, teamwork skills, collaborative behaviors, scientific creativity, doctoral students

## Abstract

Despite the growing recognition of the value of interdisciplinary learning in doctoral education, there is still a gap in the literature supporting the relationship between it and doctoral students’ scientific creativity in China. Based on a questionnaire survey of 457 doctoral students from the humanities and social sciences on the Chinese Mainland, this study adopted structural equation modeling to examine the relationships among interdisciplinary learning, teamwork skills, collaborative behaviors, and scientific creativity. The results indicated that there was a weak positive correlation between interdisciplinary learning and the scientific creativity of doctoral students. Teamwork skills mediated the relationship between interdisciplinary learning and creativity, while the mediating effect of collaborative behaviors did not hold. Moreover, the relationship between interdisciplinary learning and creativity can also be mediated by the sequential mediation of teamwork skills and collaborative behaviors.

## 1. Introduction

Higher education institutions play a crucial role in cultivating creativity, which has been increasingly recognized in both academic study and educational practices [1]. As the highest level of academic education, doctoral education strives to enhance students’ creativity [2]. Creativity, considered a critical precursor to innovation, is becoming increasingly essential for advancing knowledge and scientific progress [3]. In the academic context, fostering the creativity of doctoral students is not only vital for producing novel research, but also for equipping scholars with the skills needed for problem solving in complex research settings. Although the connotations of creativity vary in different contexts, it can generally be defined as the generation of original and effective ideas related to certain products, services, procedures, and open-ended problems [4]. Accordingly, doctoral students’ scientific creativity can be understood as the generation of novel and original ideas or solutions in academic research tasks, which contributes to breakthrough progress in their particular academic fields.

Interdisciplinary learning, in particular, has emerged as a key approach to improving learning performance, developing creativity, and generating innovative outcomes [5]. Mansilla and Duraising (2007) defined interdisciplinarity as the integration of knowledge or thinking modes from two or more disciplines [6]. Accordingly, interdisciplinary learning for doctoral students is also the process of integrating multidisciplinary knowledge, theories, or methods to promote innovation and generate creative ideas. Given that doctoral students often need to address complex academic problems, interdisciplinary learning might be effective at this stage to drive scientific creativity [7]. Several studies have highlighted the positive impact of interdisciplinary learning on students’ ability to think critically, solve complex problems, and generate innovative ideas [8,9]. At present, universities are restructuring to develop cross-disciplinary, problem-focused projects to cultivate student creativity [10].

Despite the growing recognition of the benefits of interdisciplinary learning [11], there is still a lack of empirical evidence which can provide a comprehensive explanation regarding the relationship between interdisciplinary learning and scientific creativity of doctoral students in China. Previous studies have demonstrated that doctoral students’ creativity is associated with environmental factors, such as supervisory support [12,13], funding mechanism [14], political and economic agenda [15], and group collaborative learning [16]. More importantly, creativity is not solely a social phenomenon; individual factors (such as traits, emotions, behaviors, etc.) also play a significant role, and this deserves more attention. For instance, research stress [17], creative self-efficacy [18], psychological capital [9,19], academic engagement [19], subjective well-being [20], resilience [21], and achievement motivation [22] are correlated with individual creativity. Further, generating creativity still requires certain thinking skills and behavioral patterns [23]. Our study aims to demonstrate the conditions that promote doctoral students’ scientific creativity from the perspective of interdisciplinary learning and collaborative behaviors to complement the other factors within the research. Although there were a few studies confirming that interdisciplinary learning could promote the creativity of college students [24], there is little empirical research on how interdisciplinary learning impacts doctoral students’ creativity. In addition, existing literature has primarily focused on the direct effects of interdisciplinary learning on collaborative research skills and outcomes [25], overlooking potential mediating mechanisms that may underlie this relationship. This gap in the literature underscores the need to investigate the mediating roles of teamwork skills and collaborative behaviors between interdisciplinary learning and scientific creativity of doctoral students.

The knowledge economy requires high-performing creative teams, and creativity and collaboration in education should be studied together, not only to expand our knowledge, but for practical reasons [26]. In education, most studies still view creativity and collaboration separately [27], and there is still a lot of debate about their compatibility [28]. The current study aims to address this gap by examining the mediating roles of teamwork skills and collaborative behaviors in the relationship between interdisciplinary learning and doctoral students’ creativity.

This paper is structured as follows: the next section provides a review of the literature on the relationships among interdisciplinary learning, teamwork skills, collaborative behavior, and creativity. Following that, we present the conceptual framework and research design, including the methods used to test the proposed hypotheses. Subsequently, we present the results of the structural equation modeling path analysis and the mediation effects. Finally, we discuss the implications of our findings for theory and practice, and then suggest directions for future research.

## 2. Literature Review and Hypotheses

### 2.1. Interdisciplinary Learning and Creativity

To understand how interdisciplinary learning fosters creativity, it is crucial to clarify its distinction from related concepts, such as multidisciplinarity and transdisciplinarity. According to Choi and Pak (2006) [29], multidisciplinarity involves drawing upon knowledge from various disciplines while keeping their boundaries distinct. In contrast, interdisciplinarity goes beyond simply gathering knowledge from different fields; it seeks to analyze, synthesize, and integrate insights across disciplines to form a coordinated and cohesive whole. Transdisciplinarity extends even further by uniting natural, social, and health sciences within a humanities framework, transcending traditional disciplinary boundaries altogether. The common words used for multidisciplinary, interdisciplinary, and transdisciplinary research are additive, interactive, and holistic, respectively [29]. The degree to which these three concepts cross disciplinary boundaries while at the same time interacting with each other is increasing.

Interdisciplinary learning, therefore, entails not only the acquisition of knowledge from multiple domains but also the ability to integrate and apply this knowledge in ways that enhance understanding and stimulate creativity. For doctoral students engaged in interdisciplinary studies, this approach demands both analytical and integrative skills to generate new insights and innovative solutions. Recognized as an effective strategy for stimulating individual creativity [5], interdisciplinary learning also fosters greater innovation within team settings [30], underscoring its value in both individual and collaborative contexts.

Some evidence suggested that there may be a positive correlation between interdisciplinary learning and the scientific creativity of doctoral students. Interdisciplinary learning not only unleashes students’ untapped potential, but also enhances their cognitive functions such as originality, fluency, and flexibility in thinking [31]. Moreover, interdisciplinary learning experiences could equip doctoral students with diverse knowledge and skills, which may help them to deploy their rich psychological and social resources to generate creative ideas in scientific research [32]. There have been several studies indicating that interdisciplinary team learning significantly enhances creativity, as members from different disciplinary backgrounds could bring diverse knowledge and perspectives to stimulate more innovative ideas [33,34,35]. Holley (2009) also pointed out that interdisciplinarity had a high potential to generate innovative knowledge due to the integration of diverse types of knowledge [36]. By comparing several interdisciplinary projects, Van der Wende (2007) also found that doctoral students involved in interdisciplinary learning performed significantly better than those studying a single discipline [37]. This superior performance is attributed to their ability to integrate and apply knowledge or methods from different discipline [38]. Similarly, a study by Wagner et al. (2011) indicated that interdisciplinary learning significantly improved the quality of research productivity among doctoral students [39]. Therefore, Hypothesis 1 is presented as follows.

**H1:**  *There is a significant and positive correlation between interdisciplinary learning and scientific creativity of doctoral students.*

### 2.2. Teamwork Skills as Mediator

Teamwork skills were summarized as the ability of individuals in a team to communicate and collaborate with each other in a constructive and effective manner, so as to unite and work together to achieve a common goal or complete projects, which usually helps students to integrate their work efforts and ultimately achieve teamwork success [40]. For doctoral research activities, this study defines teamwork skills as the ability of doctoral students to communicate effectively with collaborators, and strive to collaborate with other team members to complete research projects or papers.

Each disciplinary tradition has its own paradigms, basic concepts, and common terminology, so teamwork skills such as communication and understanding cultural differences are particularly important in interdisciplinary learning [41]. Some empirical studies have shown that the teamwork skills of doctoral students can be developed in interdisciplinary learning. For example, Borrego (2010) found that interdisciplinary learning could bring many positive benefits, including expanding disciplinary foundations, developing teamwork skills, and improving communication skills [42]. In the process of interdisciplinary learning, students from different disciplines need to establish effective communication and mutual understanding, which to some extent enhance their teamwork skills. Although there may be different opinions and voices in interdisciplinary projects, this learning approach that breaks down disciplinary barriers provides doctoral students with the opportunity to apply their research skills and interpersonal skills, which they consider to be unique skills valuable to their groups [25].

In addition, scientific research innovation can often not be achieved by a single person, but usually requires teamwork. Team members must engage in active and effective communication and collaboration, so as to generate new ideas and then translate them into practical actions to improve work methods, products, and services [43,44]. Kozlowski and Ilgen (2006) found that members with strong teamwork skills were more likely to play an active role in interdisciplinary learning and collaboration, driving research progress and creativity [45]. Hero et al. (2017) also believed that effective teamwork skills could help promote knowledge sharing and integration among teams, thereby enhancing individual creativity [46]. A meta-analysis by Marlow et al. (2018) further demonstrated that teamwork and communication could exert a significant positive effect on innovative outcomes [47]. The above evidence seems to indicate a close relationship between teamwork skills and creativity.

To sum up, when doctoral students engage in interdisciplinary learning, they gain more opportunities to interact with other researchers and sharpen their teamwork skills, and such collaborations further contribute to motivating their creativity. As Chang et al.’s (2022) study suggested, interdisciplinary and problem-based learning projects could improve students’ teamwork skills and then ignite scientific creativity [24]. Based on the above analysis, this study proposes Hypothesis 2.

**H2:**  *Teamwork skills play a mediating role between interdisciplinary learning and doctoral students’ scientific creativity.*

### 2.3. Collaborative Behaviors as Mediator

Collaboration can be defined as the act of working together with one or more individuals towards a common goal [48]. For doctoral students, collaborative behaviors were essentially a collective contribution that depended on the level of participation and effort of all group members in collaborative learning activities [49]. In previous studies, widespread attention was paid to the relationship between interdisciplinary learning and collaborative research behaviors among doctoral students. Borrego and Newsander (2010) argued that the clearest outcome of engaging in interdisciplinary learning is the improvement of students’ teamwork skills [42]. This is consistent with the viewpoint of Hart’s (2019) study emphasizing that interdisciplinary learning environments often foster collaborative behaviors [50]. Generally, interdisciplinary learning exposes students to the diversity of thinking and helps them to better understand different perspectives of others in a team, thereby expediting the occurrence of collaborative behaviors [51].

Moreover, the positive correlation between collaborative behaviors and creativity has also been explored by some scholars. Collaborative behaviors are conducive to the establishment of a culture of trust and collaboration among team members, thus facilitating individual creativity [52]. Astutik et al. (2020) constructed a collaborative learning model, suggesting that collaborative behaviors were effective in improving students’ creativity in science [53]. Researchers have also demonstrated that the experience of students writing papers with their teachers from the perspective of a collaborative process facilitated the acquisition of research skills and thus promoted creativity [54,55]. From the perspective of knowledge acquisition from collaborative behaviors, collaboration in interdisciplinary teams is more conducive to heterogeneous groups coming together, and expanding the scope of individual’s exploration, updating and adding new elements to their knowledge, which in turn allows them to show a greater potential for creativity [4,56]. Similarly, Li and Liu (2016) stressed the importance of knowledge diversity in the collaborative process to create synergies between different types of knowledge by strengthening linkages, ultimately leading to innovation and improved performance [57]. Thus, this study proposes Hypothesis 3:

**H3:**  *Collaborative behaviors play a mediating role between interdisciplinary learning and doctoral students’ scientific creativity.*

### 2.4. The Sequential Mediation of Teamwork Skills and Collaborative Behaviors

Previous studies have suggested that individuals with strong teamwork skills typically exhibit better collaborative behaviors, such as high-frequency information sharing, higher levels of mutual support, and stronger problem-solving abilities [58,59,60]. When doctoral students have good teamwork skills in academic research, they will be able to actively and efficiently share research interests, professional knowledge, and technical methods, thereby improving the quality of collaboration and research outcomes [25]. In a word, the interdisciplinary learning process equips students with experience in handling dissenting opinions and teamwork skills to optimize their collaborative behaviors, thereby resulting in the cultivation of creativity [61]. All of the above analyses supported the current study to propose Hypothesis 4.

**H4:**  *Teamwork skills and collaborative behaviors play the sequential mediating roles between interdisciplinary learning and scientific creativity of doctoral students.*

### 2.5. Theoretical Framework

**Figure 1:**
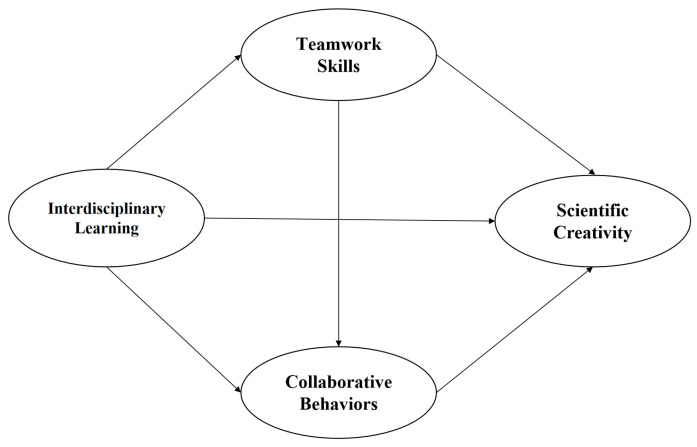
The hypothesized framework.

Cultural and social psychology recognizes social interaction, communication, and collaboration as the key elements of creativity [62]. Interdisciplinary learning, teamwork skills, and collaborative behaviors, which are the focus of this study, all contain elements that can explain the formation of creativity. According to Social Cognitive Theory, individuals learn from observing others and develop their skills through wide social interactions [63]. From this perspective, interdisciplinary learning can provide doctoral students with opportunities to observe and learn from others with different expertise, which contributes to enhancing their teamwork skills and facilitating the occurrence of cooperative behaviors, thereby promoting their creativity [64]. In another word, interdisciplinary learning can enhance doctoral students’ ability to work in teams, which in turn facilitates their collaborative behaviors, and further contributes to the enhancement of creativity [65]. Therefore, teamwork skills and collaboration behaviors create a chain of mediating effects between interdisciplinary learning and scientific creativity of doctoral students. In this study, the hypothesized framework is presented in Figure 1.

## 3. Methods

### 3.1. Samples

This study collected questionnaire data from doctoral students in the humanities and social sciences on the Chinese mainland. Considering the stratification of the Chinese higher education system, the stratified random sampling method was used to investigate the doctoral students at research universities. We contacted 4 teachers and 9 students from different levels of universities through our personal network, and requested their assistance in spreading the questionnaire on a larger scale through online platforms (such as We-Chat or email). Before filling in the question items, all participants were informed that these data were anonymous and only used for research purposes in the form of questionnaire guidance to reassure respondents to provide their true feelings. After data cleaning and the elimination of invalid questionnaires, a total of 457 valid questionnaires were finally collected.

The demographic data of the participants are shown as follows. Among them, 58.2% (n = 266) were male and 41.8% (n = 191) were female. In terms of grade, 56.0% (n = 256) of respondents were from the first to second year of their doctoral research, 31.5% (n = 144) were from the third to fourth year, and 12.5% (n = 57) were recent holders of a doctoral degree. In terms of disciplines, those in the humanities (literature, history, and philosophy) accounted for 15.1% (n = 69), while the social sciences (economics, management, law, education, and society) accounted for 84.9% (n = 388). At the university level, the Chinese higher education system has undergone changes from the “211 Project” and “985 Project” to a “Double First-class Construction”. The current hierarchical structure of higher education in China can be specifically divided into three levels or tiers, ranked from the highest to the lowest level in order of First-class universities, First-class Discipline universities, and then non-Double First-Class universities [66]. In this study, 56.7% (n = 259) were from First-class universities, 26.7% (n = 122) were from First-class Discipline universities, and 16.6% (n = 76) were from non-Double First-Class universities.

### 3.2. Instruments

This study incorporated four scales, including the Interdisciplinary Learning Scale, Creativity Scale, Teamwork Skills Scale, and Collaborative Behaviors Scale. All items were scored using a 7-point Likert Scale, ranging from “I strongly disagree” to “I strongly agree”. The higher the degree of agreement expressed by the respondents, the higher their self-reported scores in that dimension. Table 1 presents the result of a confirmatory factor analysis for all scales. The average of variance extracted (AVE), an evaluation of the convergent validity of the measurement model, was used to calculate the average explanatory power of each measurement item for latent variables. Bagozzi (1981) suggested that an AVE greater than 0.5 was best, with 0.36~0.5 being an acceptable threshold [67]. In our study, the AVE values ranged from 0.504 to 0.699, showing a good convergent validity. In addition, Hair et al. (2011) recommended that composition reliability (CR) values for the constructs should exceed the cutoff value of 0.7 [68]. In our study, the CR values ranged from 0.835 to 0.921, proving the high internal reliability of our results.

*Interdisciplinary Learning Scale*. Misra et al.’s (2009) study designed an 8-item scale (Chronbach’s alpha = 0.843) to measure undergraduate interdisciplinary learning, such as participating in learning groups with researchers in multiple fields, designing a new collaborative study, and taking classes outside one’s major [69]. Then, Keck et al. (2017) further developed these indicators for measuring the interdisciplinary training of PhD students [70]. This scale consisted of 5 items to investigate doctoral students’ behaviors in participating in interdisciplinary courses, interdisciplinary research projects, and reading interdisciplinary journals. Specifically, the sample items are as follows: “Take class outside your field or major”, “Read journals outside your field or major”, “Involved in interdisciplinary research projects”, and “Participate in interdisciplinary forums or lectures”, etc. The standardized factor loading of all latent variables was significant (*p* < 0.001, β = 0.802–0.882), SMC = 0.643–0.778, CR = 0.921, the AVE was 0.699, and its Chronbach’s alpha was 0.920.

*Creativity Scale*. This scale originated from Madjar et al.‘s (2011) scale on creativity [71], which was developed by Tang and Ding (2014) [72] and Yao et al. (2024) [73] to measure graduate students’ creativity, with a good reliability in the Chinese context. Based on them, and considering that doctoral students are mainly engaged in academic research activities, our creativity scale included 4 items, such as “I can use knowledge from other fields to develop new ideas”, “I can come up with innovative research questions”, “I come up with practical ideas to improve research performance” and “I often have new ideas to improve the efficiency of my research”. The standardized factor loading of all latent variables was significant (*p* < 0.001, β = 0.792–0.840), SMC = 0.627–0.706, CR = 0.886, AVE = 0.660, Chronbach’s alpha = 0.885.

*Teamwork Skills Scale*. This scale was designed mainly based on Adair et al.’s (2013) Team Cultural Intelligence Scale, which paid special attention to communication, coordination, and working effectively in diverse teams [74], providing validated instruments that could be used for measuring the teamwork skills of doctoral students. We also referred to the items in the three dimensions (conflict management, communication, problem solving) of the Teamwork Competency Scale (TCS) developed by Hebles et al. (2022) [75]. Finally, our scale included 3 items, such as “I am sensitive to and open to cultural differences in cooperation”, “I can apply what I have learned to interpersonal communication”, and “I can work effectively in a team”. The standardized factor loading of all latent variables was significant (*p* < 0.001, β = 0.814–0.847), SMC = 0.663–0.717, CR = 0.866, AVE = 0.683, Chronbach’s alpha = 0.864.

*Collaborative Behaviors Scale*. According to the division of tasks in scientific research activities, collaborative research behaviors among doctoral students can be distilled into multiple types, including discussion on the selection of research questions, data collection, data analysis, and project management, etc. [76], which provides a comprehensive picture of the different roles or contributions of doctoral students in research collaboration. Based on it, our scale was constructed with 5 items, including developing ideas and research plans, collecting data, analyzing research data, writing discussions and conclusions, and submitting and revising the manuscript, with a Chronbach’s alpha of 0.834. The higher their reported scores on these items, the deeper the degree of their participation in scientific research collaboration. The standardized factor loading of all latent variables was significant (*p* < 0.001, β = 0.674–0.751), SMC = 0.454–0.564, CR = 0.835, AVE = 0.504.

### 3.3. Data Analysis

In this study, SPSS 25 and AMOS 24 were used for data analysis. First, descriptive statistics were conducted using SPSS 25 to calculate the mean and standard deviation of each dimension, and the Pearson correlation test was adopted to analyze the correlation between interdisciplinary learning, teamwork skills, collaborative behaviors, and the scientific creativity of doctoral students. Second, a structural equation model was constructed to examine the path relationship among these four variables. Based on 5000 random repeated samples, we employed the deviation-corrected non-parametric percentile Bootstrap method to determine the 95% confidence interval of the mediating effect, thereby examining the single mediation and sequential mediation of teamwork skills and collaborative behaviors on the relationship between interdisciplinary learning and creativity of doctoral students.

## 4. Results

### 4.1. Common Method Bias Test

Harman’s single-factor test is considered to be the most widely adopted technique for detecting common method biases [77,78]. If an unrotated solution (including all measurement items) yields a single factor that accounts for more than 50% of the variance, there is a common method bias [77]. In this study, the result indicated that the single factor accounted for 48.53% of the explained variance. Therefore, common method bias does not appear to be a significant concern in this study.

### 4.2. Descriptive Statistics and Correlation Analysis

Table 2 presents the descriptive statistics of the variables and the correlation test results. Interdisciplinary learning was significantly and positively correlated with teamwork skills (r = 0.579, *p* < 0.01), collaborative behaviors (r = 0.435, *p* < 0.01), and doctoral students’ scientific creativity (r = 0.568, *p* < 0.01). Teamwork skills (r = 0.752, *p* < 0.01) and collaborative behaviors (r = 0.592, *p* < 0.01) were significantly and positively correlated with creativity. In addition, there was a significant positive relationship between teamwork skills and collaborative behaviors (r = 0.538, *p* < 0.01). The AVE square root values on the diagonal in Table 2 are all greater than the correlation coefficients between the variables, indicating that these constructs have good convergent and discriminant validity. It implies that all the variables measured by the scales in our study are relatively independent, and each can effectively reflect its underlying concept. The correlation test results supported a further examination of Figure 1 using structural equation model.

### 4.3. Model Fit Indices

Table 3 shows the recommended values for each indicator of the model fitting index, as well as the actual values in our study, indicating that the data conformed well to the hypothetical model (X2 = 318.989, df = 113, X2/df = 2.823, CFI = 0.959, TLI = 0.950, RMSEA = 0.063). Specifically, for each indicator, the Chi-Square value relative to the degree of freedom (X2/Df) is less than the cut-off value of 3.00, suggesting an acceptable fit [79]. The comparative fit indices (CFI) of 0.959 indicated a good fit [80]. The Tucker–Lewis index (TLI) values were above 0.9, suggesting a good fit. Meanwhile, the root mean square error of approximation (RMSEA) was also acceptable since it was below the threshold of 0.08 [68].

### 4.4. Path Test of Structural Equation Model

Table 4 presents the mutual relationships among these variables including interdisciplinary learning, teamwork skills, collaborative behaviors, and creativity. As shown in Table 3, the estimate is the non-standardized path coefficient, S.E. is used to estimate the standard error of parameters, and C.R. is the t-value of the t-test. If the C.R. value is greater than 1.96, it indicates a significance level of 0.05.

**Figure 2:**
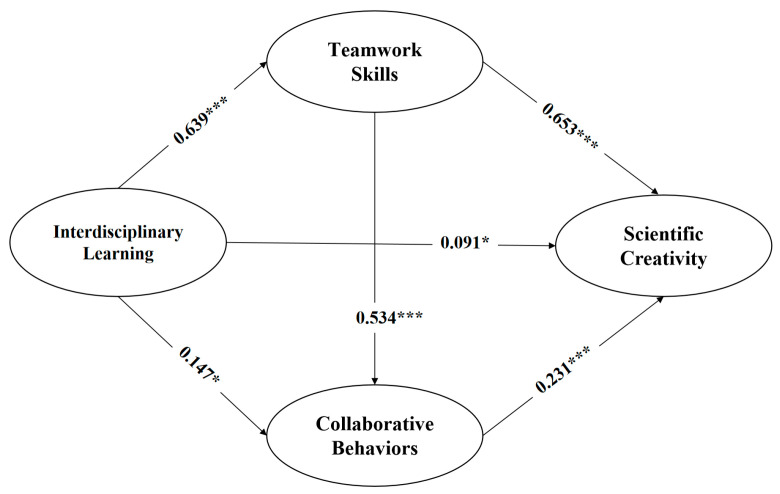
Structural equation model result. Note: *** *p* < 0.001; * *p* < 0.05.

In Figure 2, the path coefficient suggested that there was a significant and weak positive relationship between interdisciplinary learning and the scientific creativity of doctoral students (β = 0.091, *p* < 0.05), so H1 was supported. Moreover, interdisciplinary learning were also shown to have a significant positive effect on teamwork skills (β = 0.639, *p* < 0.001) and collaborative behaviors (β = 0.147, *p* < 0.05). Teamwork skills (β = 0.653, *p* < 0.001) and collaborative behaviors (β = 0.231, *p* < 0.001) also exerted a significant positive effect on doctoral students’ creativity. In addition, there was a significant positive relationship between teamwork skills and collaborative behaviors (β = 0.534, *p* < 0.001). Whether the single mediation or sequential mediating effects of teamwork skills and collaborative behavior are tenable needs further testing.

### 4.5. Mediation Effect Test

The results of the mediation effect test are shown in Table 5. The estimated total effect value of interdisciplinary learning on doctoral students’ creativity was 0.443 (Z > 1.96, 95% CI = [0.333, 0.549]), and the total indirect effect value was 0.378 (Z > 1.96, 95% CI = [0.270, 0.486]). Through the calculation, it was found that the total indirect effect accounted for 85.3% of the total effect of interdisciplinary learning on creativity.

In addition, the results shown in Table 5 further illustrate the mediating effects of teamwork skills and collaborative behaviors. First, the single mediation of teamwork skills was significant (Z > 1.96, *p* < 0.05, 95% CI = [0.190, 0.449]), accounting for 67.3% of the total effect, and the point estimate was 0.298, so H2 was supported. Second, the mediation of collaborative behaviors accounted for 5.4% of the total effect, and the point estimate was 0.024, but the mediating effect between interdisciplinary learning and creativity was not significant, so H3 was rejected. Third, the sequential mediation of teamwork skills and collaborative behaviors accounted for 12.6% of the total effect, and the point estimate was 0.056, with a significant mediating effect (Z = 2.800, *p* < 0.05, 95% CI = [0.022, 0.098]), so H4 is supported.

In summary, in the relationship between interdisciplinary learning and scientific creativity, the result supported the single mediation of teamwork skills, and the chain of mediating effects of teamwork skills and collaborative behaviors, but rejected the single mediation of collaborative behaviors. Meanwhile, the separate mediation effect of teamwork skills was stronger than the dual mediating impact of teamwork skills and collaborative behaviors.

## 5. Discussion

First, this study found that interdisciplinary learning had a weak positive effect on doctoral students’ creativity, indicating that encouraging doctoral students to engage in interdisciplinary learning could more or less stimulate their scientific creativity. Our findings are consistent with the conclusion of Van der Meer et al.’s (2010) study [81], which suggested that doctoral students involved in interdisciplinary projects exhibited a higher level of scientific creativity during the academic research process. This might because interdisciplinary learning not only exposes students to different perspectives and methodologies, but also encourages them to integrate knowledge from multiple disciplines to understand and solve complex problems [82], thereby enhancing their scientific creativity and capacity for innovation [64,83]. Edmondson and Harvey (2018) also described many benefits of interdisciplinary learning, and indicated that it was an important measure for generating innovation and scientific creativity [5]. For example, interdisciplinary learning helps students to acquire diverse perspectives, analyze broad and complex backgrounds, create more innovative ideas, and expand the range of innovative perspectives that they can utilize when working in a team [5]. Conversely, more creative students might engage more in collaborative activities and participate in more interdisciplinary projects. To sum up, interdisciplinary learning is crucial for the scientific creativity of doctoral students, and this finding may provide empirical evidence for carrying out interdisciplinary learning and training in doctoral education practices.

Second, our study revealed the mediating role of teamwork skills in the relationship between interdisciplinary learning and scientific creativity, demonstrating that the ability to work effectively in a team is a critical factor that benefits the creative outcomes of interdisciplinary learning experiences. There are several potential reasons that might explain this mediating mechanism. On the one hand, teamwork skills facilitate better communication among group members, allowing for the wide exchange of diverse ideas and perspectives from multiple disciplines. In interdisciplinary settings, the integration of knowledge from different fields can lead to innovative solutions and creative research outcomes [84]. On the other hand, effective teamwork skills include the ability to manage and resolve conflicts, which may arise from the collision of different disciplinary perspectives. By navigating these conflicts constructively, teams can synthesize diverse viewpoints into cohesive and creative research ideas. In addition, good teamwork skills also involve coordinating efforts and managing project tasks efficiently, which ensures that interdisciplinary projects are well organized and meet deadlines, and that collaborative efforts align with creative research goals. In another word, teamwork skills can help teams optimize the rigor, interdisciplinarity, feasibility, and viability of developing creative research outcomes [85]. Therefore, doctoral students’ engagement in interdisciplinary learning might cultivate their scientific creativity, largely through the mediating role of teamwork skills.

Third, the mediating effect of collaborative behaviors between interdisciplinary learning and scientific creativity is not significant, which may be related to the differences in research activities among doctoral students in different disciplines, as well as the difficulty of scientific cooperation. On the one hand, in natural and engineering sciences, innovative research and creativity cultivation rely on specific laboratories based on the faculty [86], reflecting a unique “laboratory culture”, which is relatively rare in the humanities and social sciences. Compared to them, those doctoral students in humanities and social sciences do not need to depend entirely on laboratories, and their creativity and innovative research achievements are often the result of individual efforts and explorations. Moreover, interdisciplinary learning exposes them to knowledge, perspectives, and methods from multiple disciplines, and their integration of them can be conducive to their creativity, rather than necessarily through collaborative behaviors. On the other hand, interdisciplinary learning involves knowledge systems from multiple disciplines, and this complexity may increase the difficulty of communication and coordination during collaborative processes, thereby affecting the effectiveness of collaborative behaviors. Moreover, regarding learning preference differences, due to individual preferences for personalized or collaborative learning [45], collaborative behaviors may not significantly mediate the relationship between interdisciplinary learning and creativity for those who prefer to learn alone.

Finally, the chain of mediating effects of teamwork skills and collaborative behaviors between interdisciplinary learning and creativity has been tested. Interdisciplinary learning contributes to enhancing doctoral students’ teamwork skills, as they will come into contact with different people during interdisciplinary communication and learn to collaborate with others on scientific research [61]. These trained teamwork skills can help them become more effective in coordinating and integrating the different opinions of team members [60]. Generally, those doctoral students with good teamwork skills are often not afraid of criticism, express their ideas without hesitation, and actively engage in scientific research cooperation with others. Teamwork skills such as active listening, conflict resolution, and mutual support can also improve the quality of collaborative behaviors, thereby stimulating more original ideas and higher creative performances in the collision of thoughts [87]. In addition, in the sequential mediation model, the teamwork skills of doctoral students help create a harmonious team collaboration atmosphere where iterative feedback and continuous improvement are highly valued. Through multiple iterations and feedback, the quality of research collaboration behaviors among doctoral students can be effectively improved, resulting in more refined and creative research outcomes [88].

## 6. Contributions and Implications

Throughout history, multidisciplinary scholars like Leonardo da Vinci have inherently embodied many of the very principles that underlie creativity [89]. Snow (1959) emphasizes that interdisciplinary learning and collaboration could yield creative breakthroughs [90], but also that different educational and cultural systems reinforce specialization and maintain this divide, making integration challenging. Nowadays, the persistent existence of complex global issues, such as climate change, poverty, and sustainability, highlights the limitations of knowledge constrained by traditional disciplinary boundaries [7]. Interdisciplinarity has been an intellectual paradigm viewed as essential for universities to foster students’ creativity so as to tackle complex issues that span disciplinary boundaries, with a growth of interdisciplinary doctoral programs [36]. Further, despite the growing emphasis on interdisciplinary doctoral learning and training, there is little academic evidence for this initiative. Therefore, the purpose of this study is to provide new perspectives and evidence to support the role of interdisciplinary learning in fostering doctoral students’ scientific creativity.

This study untangled the relationships among interdisciplinary learning, teamwork skills, collaborative behaviors, and the scientific creativity of doctoral students in China, which provides several theoretical implications. First, although interdisciplinary experience has been considered an important potential factor in shaping students’ creativity, there was little empirical research on the relationship between interdisciplinary learning and creativity during doctoral education. Rooted in the context of the rapidly expanding number of Chinese doctoral students, the current study widens our understanding of the influencing factors affecting doctoral students’ creativity from the perspective of individual learning processes, such as interdisciplinary learning, teamwork skills, and collaborative behaviors. Second, we constructed a model of the mechanism behind the influence of interdisciplinary learning on scientific creativity, and then validated the separate mediation of teamwork skills and the sequential mediation of teamwork skills and collaborative behaviors, enriching the existing impact mechanism framework. Third, several existing studies focused only on the direct influences of contextual factors on doctoral students’ creativity, overlooking the microscopic psychological and behavioral processes [9]. This study started from the micro perspective which is most close to the learning process of doctoral students, and proved the key roles of individual interactive abilities (such as teamwork skills) and behaviors (such as collaborative behaviors) in shaping creativity, calling on academia to pay attention to the micro factors in creativity cultivation rather than simply focusing on external contexts.

We have also derived some inspiration for important practical considerations from these findings. On the one hand, the result indicated that interdisciplinary learning had a positive direct and indirect impact on the creativity of doctoral students, which demonstrates that the integration of disciplines triggers innovative potential. Therefore, university administrators can consider interdisciplinary education as a direction to promote scientific research innovation. In the practice of doctoral education, different departments should be encouraged to collaborate on interdisciplinary curriculum design, and doctoral students should be supported to participate in interdisciplinary projects and lectures, absorbing and applying professional knowledge and technologies from different disciplinary fields. On the other hand, this study suggested that teamwork skills and collaborative behaviors played a mediating role between interdisciplinary learning and creativity. This should inspire university faculty members to cultivate doctoral students’ social skills and teamwork abilities when conducting interdisciplinary project teaching.

## 7. Limitations

There are several limitations that should be improved in future research. First, we collected cross-sectional data, and so it may be difficult to capture the changes in the contribution of interdisciplinary learning to creativity over time and its causal relationship. Therefore, we recommend using a longitudinal design to collect multi-period tracking data or an experimental design to evaluate the causal effect among these variables. Second, a reliance on self-reported data may bring potential challenges, including biases related to social desirability and the absence of objective personality assessments, which may potentially inflate the associations observed in these findings. Due to the limited number of items measured in the questionnaire, and the complex connotation of scientific creativity in doctoral education, more and more possibilities have emerged for how to evaluate creativity. In the future, more comprehensive measures for other traits (such as situational testing), and more standardized measures, such as ones tapping into divergent thinking (like the alternative uses task) or convergent thinking tasks (like the remote associations tasks), can be adopted to reflect potential psychological mechanisms. Third, being constrained by the questionnaire collection, we only focused on humanities and social sciences doctoral students. Considering the differences in scientific research among doctoral students majoring in the humanities, social sciences, natural sciences, and engineering, we look forward to seeing more research investigating doctoral students involved in science and engineering, so as to compare the impact mechanisms between the interdisciplinary learning and creativity of these two groups.

## 8. Conclusions

This study found that there was a significant positive correlation between interdisciplinary learning and doctoral students’ scientific creativity. Meanwhile, the single mediation of teamwork skills in the effect of interdisciplinary learning on scientific creativity was revealed, while the single mediating role of collaborative behaviors was not supported. Moreover, the sequential mediation of teamwork skills and collaborative behaviors also held true. Our results imply that interdisciplinary learning might first improve doctoral students’ teamwork skills, which promotes their effective collaborative behaviors and ultimately benefits their scientific creativity.

## Figures and Tables

**Table 1:** Confirmatory factor analysis.

Dimensions	Items	Z	Item Reliability		CR	AVE
			Std	SMC		
Interdisciplinary Learning	5	21.986–24.710	0.802–0.882	0.643–0.778	0.921	0.699
Teamwork Skills	3	19.389–20.392	0.814–0.847	0.663–0.717	0.866	0.683
Collaborative Behaviors	5	12.516–13.562	0.674–0.751	0.454–0.564	0.835	0.504
Scientific Creativity	4	19.724–20.581	0.792–0.840	0.627–0.706	0.886	0.660

**Table 2:** Means, standard deviations, and correlations among variables.

Variable	1	2	3	4
1. Interdisciplinary Learning	**0.837**			
2. Teamwork Skills	0.579 **	**0.827**		
3. Collaborative Behaviors	0.435 **	0.538 **	**0.711**	
4. Scientific Creativity	0.568 **	0.752 **	0.592 **	**0.802**
Mean	5.337	5.581	5.712	5.688
Standard Deviation	1.292	1.120	0.999	1.009

Note: ** Significant correlation at the 0.01 level (two-tailed); the bold value is the root value of AVE, as a measure of discriminant validity.

**Table 3:** Model fit indices.

Fit Indices	Suggested Value	Model	Fitting Degree
ML X2	Relatively small	318.989	—
Df	Relatively large	113	—
X2/Df	1 < X2/Df < 10	2.823	passed
CFI	>0.9	0.959	passed
TLI	>0.9	0.950	passed
RMSEA	<0.08	0.063	passed

**Table 4:** Structural equation model path coefficients.

IV		DV	Estimate	S.E.	C.R.	*p*	Std.
Interdisciplinary Learning	→	Teamwork Skills	0.496	0.038	12.928	***	0.639
Interdisciplinary Learning	→	Collaborative Behaviors	0.096	0.041	2.349	0.019	0.147
Teamwork Skills	→	Collaborative Behaviors	0.447	0.060	7.426	***	0.534
Collaborative Behaviors	→	Scientific Creativity	0.253	0.054	4.663	***	0.231
Teamwork Skills	→	Scientific Creativity	0.600	0.057	10.567	***	0.653
Interdisciplinary Learning	→	Scientific Creativity	0.065	0.032	2.003	0.045	0.091

Note: Std means standardized path coefficient, IV means independent variable, DV means dependent variable; *** *p* < 0.001.

**Table 5:** Mediating effect test.

Mediation Effect	Point Estimate	Product of Coefficients		Bootstrapping 95% CI				*p*	Mediation Effect Ratio
				Bias Corrected		Percentile			
		SE	Z	Lower	Upper	Lower	Upper		
Total effect	0.443	0.053	8.358	0.333	0.549	0.344	0.554	-	-
Direct effect	0.065	0.051	1.275	−0.025	0.184	−0.040	0.179	-	-
Indirect effect	0.378	0.058	6.517	0.270	0.486	0.294	0.513	0.025	85.3%
Indirect effect (IL-TS-SC)	0.298	0.067	4.448	0.190	0.449	0.190	0.460	0.012	67.3%
Indirect effect (IL-CB-SC)	0.024	0.021	1.143	−0.003	0.078	−0.003	0.083	0.130	5.4%
Indirect effect (IL-TS-CB-SC)	0.056	0.020	2.800	0.022	0.098	0.022	0.098	0.010	12.6%

Note: SE: standard error; IL: interdisciplinary learning; TS: teamwork skills; CB: collaborative behaviors; SC: scientific creativity.

## Data Availability

The anonymized data that support the findings of this study are available on request from the corresponding author. The data are not publicly available due to containing information that may comprise the participants’ privacy.
